# AXL Receptor in Breast Cancer: Molecular Involvement and Therapeutic Limitations

**DOI:** 10.3390/ijms21228419

**Published:** 2020-11-10

**Authors:** Italia Falcone, Fabiana Conciatori, Chiara Bazzichetto, Emilio Bria, Luisa Carbognin, Paola Malaguti, Gianluigi Ferretti, Francesco Cognetti, Michele Milella, Ludovica Ciuffreda

**Affiliations:** 1 Medical Oncology 1, IRCCS-Regina Elena National Cancer Institute, 00144 Rome, Italy; fabiana.conciatori@ifo.gov.it (F.C.); chiara.bazzichetto@ifo.gov.it (C.B.); paola.malaguti@ifo.gov.it (P.M.); gianluigi.ferretti@ifo.gov.it (G.F.); francesco.cognetti@ifo.gov.it (F.C.); 2 U.O.C. Medical Oncology, Fondazione Policlinico Universitario Agostino Gemelli, IRCCS, Università Cattolica del Sacro Cuore, 00168 Rome, Italy; emilio.bria@unicatt.it; 3 Department of Woman & Child Health, Division of Gynecologic Oncology, Fondazione Policlinico Universitario Agostino Gemelli IRCCS, 00168 Rome, Italy; luisa.carbognin@gmail.com; 4 Section of Oncology, Department of Medicine, University of Verona School of Medicine and Verona University Hospital Trust, 37126 Verona, Italy; michele.milella@univr.it; 5 SAFU, Department of Research, Advanced Diagnostics, and Technological Innovation, IRCCS-Regina Elena National Cancer Institute, 00144 Rome, Italy; ludovica.ciuffreda@ifo.gov.it

**Keywords:** AXL, breast cancer, biomarkers, targeted therapy, immunotherapy, tumor microenvironment

## Abstract

Breast cancer was one of the first malignancies to benefit from targeted therapy, i.e., treatments directed against specific markers. Inhibitors against HER2 are a significant example and they improved the life expectancy of a large cohort of patients. Research on new biomarkers, therefore, is always current and important. AXL, a member of the TYRO-3, AXL and MER (TAM) subfamily, is, today, considered a predictive and prognostic biomarker in many tumor contexts, primarily breast cancer. Its oncogenic implications make it an ideal target for the development of new pharmacological agents; moreover, its recent role as immune-modulator makes AXL particularly attractive to researchers involved in the study of interactions between cancer and the tumor microenvironment (TME). All these peculiarities characterize AXL as compared to other members of the TAM family. In this review, we will illustrate the biological role played by AXL in breast tumor cells, highlighting its molecular and biological features, its involvement in tumor progression and its implication as a target in ongoing clinical trials.

## 1. Perspective Chapter

In the oncological field, therapeutic scenarios are multiple and have evolved considerably in recent years. Unspecific treatments, such as chemotherapy, have given way to the new pharmacological frontiers of targeted therapy and immunotherapy. The discovery of new molecular targets and the well-established evidence of the tight interconnection between tumor cells and the microenvironment have revolutionized the clinical management of patients. Human epidermal growth factor receptor 2 (HER2) or estrogen receptor and (ER)/PROGESTERONE RECEPTOR (PR)-positive tumors have benefited from targeted treatments, but particularly aggressive forms have yet to find the optimal therapeutic approach. For example, for triple-negative breast cancer (TNBC), the research for new biomarkers is always open. AXL, especially in this molecular background, performs many oncogenic functions and is considered a potential marker in which to invest. However, although a great amount of evidence confirms that AXL is a key element of breast cell tumorigenesis, its clinical implications are still limited. Many inhibitors currently in use are multi-targets, and AXL’s selective inhibitors are still in pre-clinical trials. Therefore, the necessity to better explore the molecular implications of AXL and its involvement in the immune response becomes even more important in order to develop its selective and effective molecules.

## 2. Introduction

Currently, breast cancer is the second leading cause of oncological death in the female population globally and represents about 30% of invasive tumors in women [1,2]. Although significant advances have been made in the treatment of this pathology, phenomena such as metastasis and drug resistance still remain unresolved. Indeed, even if targeted therapy and immunotherapy have greatly improved the life expectancy of many patients, they have not bypassed these problems at the basis of many cancer deaths. The use of high performance “omics” technologies, in recent years, has accelerated the identification of new molecular targets able to perform the dual function of predictive and/or prognostic factors [3]. Moreover, an extensive study of the biomarkers’ action mechanisms is desirable to allow the development of new inhibitors.

In recent years, it has emerged that receptor and non-receptor tyrosine kinases (TKs) are often overexpressed/mutated in many tumor models, including breast cancer, representing a possible molecular target in clinical cancer therapy [4]. TKs are a big family of proteins that perform a mediator role between the inside and outside of cell. Their activation regulates cell differentiation, proliferation, apoptosis and metabolism [5,6,7]. Receptor TKs (RTKs) include 20 families classifiable according to amino acid sequence within the kinase domain and structural analogism within extracellular regions [8,9,10]. The ligand/receptor interaction triggers a biochemical reaction cascade that culminates in tyrosine residue phosphorylation on different substrates [9]. RTKs’ subfamily is composed of TYRO-3 (or Sky), AXL (or UFO) and MER (or Eyk, Nym and Tyro12) (TAM) receptors, essentially formed by an extracellular region and a cytoplasmic kinase domain. Growth arrest-specific 6 (GAS6), protein S1 (PROS1), Tubby, Tubby-like protein 1 (TULP-1) and Galectin-3 are the most important ligands of TAMs [11,12]. Despite its highest affinity to AXL, GAS6 recognizes and binds TYRO-3 and MER also, while PROS1 seems to interact only with TYRO-3 and MER [12,13]. Moreover, TULP-1 binds all three TAM receptors and Tubby and Galectin-3 only recognize MER [14,15,16]. TAM receptors are overexpressed in several human malignancies, including leukemia, melanoma, breast, lung, gastric and colon cancers, promoting upregulation of pro-survival pathways [17,18]. All members of the TAM family are involved in the development and progression of different forms of cancer, such as lung and colon tumors [19,20]; however, in the genesis of the breast cancers, AXL is particularly involved; indeed, in this tumor context, it results often dysregulated and its overexpression is associated to unfavorable outcomes for patients. In addition, AXL contributes to all stages of malignant breast cell transformation, especially by affecting the transition to a mesenchymal and invasive state [21]. Therefore, several inhibitors for this receptor are developed, although its high structural affinity with other RTKs makes it difficult to produce specific drugs. BGB324 (Bemcentinib or R428) is probably the most selective AXL inhibitor and, recently, in combination with pembrolizumab, was implicated in clinical trials for triple-negative breast cancer (TNBC) and adenocarcinoma [22].

In this review, we summarize the current knowledge of AXL functions and its implications in breast cancer progression.

## 3. AXL Receptor Tyrosine Kinase

AXL (from the Greek “anexelekto” which means “uncontrolled”) is frequently overexpressed in several human cancers (such as breast, lung, gastric, metastatic colon and prostate tumors) and is associated with metastasis and poor prognosis [23]. Several studies, indeed, have shown a significant correlation between AXL expression and clinical outcome of the patients [24,25]. An example is the retrospective study conducted by Masashi Ishikawasu and collaborators on non-small-cell lung cancer (NSCLC) patients. They observed that the overall survival (OS) rate at 5 years is 77.5% and 38.6% in patients with low or high AXL levels, respectively [26]. AXL was first isolated in 1988 from patients with chronic myelogenous leukemia and chronic myeloproliferative disorders. Physiologically expressed in several organs and tissue, it is a mediator of many cellular processes, such as phagocytosis, cell migration, platelet aggregation and inflammation. As such, AXL’s aberrant expression/activation are strongly associated with tumor progression [21,27].

### 3.1. AXL Protein Structure

**Figure 1:**
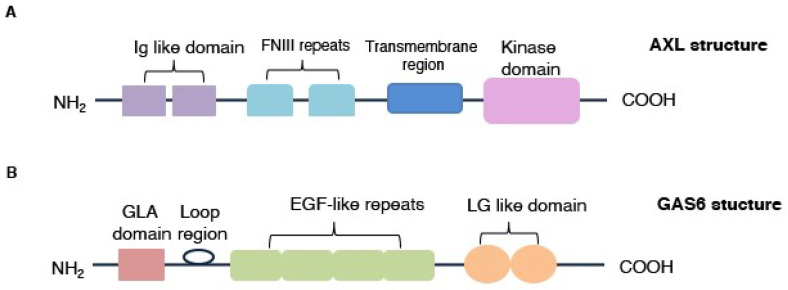
AXL receptor and GAS6 protein structures. (**A**) AXL is structurally composed by two immunoglobulin (Ig)-like domains, two fibronectin type III (FNIII) repeats, a transmembrane region and cytoplasmatic domain, implicated in kinase activity. (**B**) GAS6 is characterized by a γ-carboxyglutamic acid (Gla) domain, a loop region, four EGF-like repeats and two C-terminal globular laminin G-like (LG) domains that promote interaction with AXL.

AXL is composed of an extracellular portion with two immunoglobulin (Ig)-like domains and dual fibronectin type III (FNIII) repeats, a transmembrane region and a cytoplasmic domain, responsible for its tyrosine kinase activity (Figure 1A) [11,12,13,17,28,29]. GAS6, a 678-amino-acid protein, belongs to the vitamin K-dependent family, represents the major AXL’s ligand and is divided into an N-terminal region containing multiple gamma-carboxy-glutamic acid (Gla) residues, a loop region, four EGF-like repeats and two C-terminal globular laminin G-like (LG) domains (Figure 1B) [12,15,30].

### 3.2. AXL Expression

**Figure 2:**
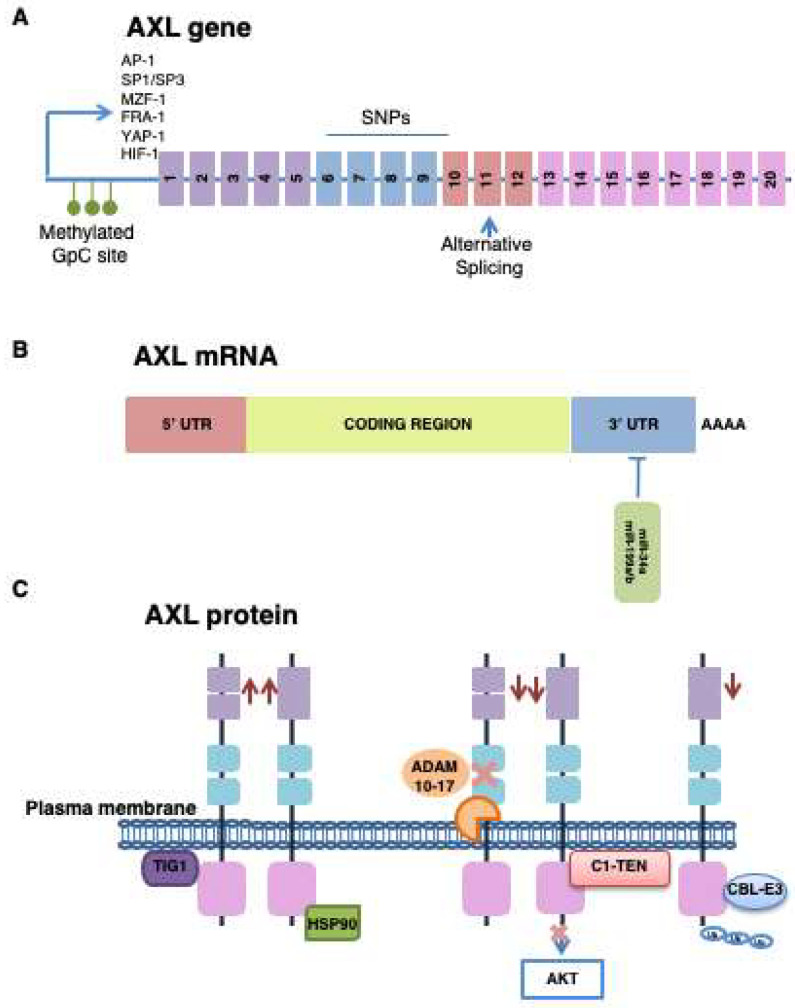
AXL expression: control mechanisms. (**A**) AXL expression is epigenetically regulated through methylation of the CpG islands present in its promoter. In contexts with high AXL levels, the promoter methylation is essentially absent. Moreover, the AXL gene promoter presents binding sites for more transcription factors. The splicing alternative, in exon 10, promotes the formation of two AXL functional variants; in addition, three single polymorphisms (SNPs), between exons 6 and 10, are identified. (**B**) miR-34a and miR-99a/b negatively regulate the receptor expression through their interaction with 3′UTR region of AXL. (**C**) Many proteins can negatively or positively regulate AXL’s expression or activity. A disintegrin and metalloproteinase (ADAM) domain containing protein 10 and 17 and casitas B-lineage lymphoma (CBL) E3 ligases are responsible for AXL inactivation and ubiquitination, respectively. C1 domain-containing phosphatase and tensin homolog (C1-TEN), inhibiting AXL catalytic domain, blocks its ability to active AKT. Heat-shock protein 90 (HSP90) and Tazarotene-induced gene 1 (TIG1), instead, stabilize AXL in plasma membrane.

AXL is a highly conserved gene between vertebrate species. It is localized on chromosome 19q13.2 and is characterized by two alternative variants as a result of splicing the site of exon 10, within the trans-membrane domain. Both variants work normally because the intra- and extracellular domains are preserved [31,32,33,34]. Tumorigenesis, in its various aspects, is influenced mainly by AXL overexpression and not by its possible genetic alterations. Indeed, very few AXL-activating mutations and genomic amplifications have been found. In breast cancer, data obtained from cBIOPortal show mutations in only 2% of cases [28]. Instead, three single polymorphisms (SNPs), within exons 6 and 10 of the *AXL* gene, have been identified (Figure 2A) [35].

#### 3.2.1. Transcriptional Regulation

Several transcription factors modulate *AXL* expression: activator protein 1 (AP-1), myeloid Zinc Finger 1 (MZF-1), transcription factor Fos-related antigen 1 (FRA-1), yes-associated protein 1 (YAP-1) and specificity protein (SP)1/3 [36,37,38,39]. Mudduluru G. and collaborators have emphasized, in different cellular contexts, the key role of SP1/SP3 in the correct basal expression of AXL; silencing or upregulation of these transcriptional factors deeply modulates the receptor expression [37]. Moreover, 19 CpG islands, within and around the SP-binding site, influence the interaction between SP1 and AXL promoter. The methylation of these CG-rich regions is an important epigenetic mechanism to block AXL transcription. Indeed, in colon cancers analyzed, CpG islands resulted partially methylated in cells with low AXL expression, as compared to cells with the highest AXL levels [37]. Microenvironment factors can also determine changes in AXL expression. Several studies have shown that upregulation of hypoxia-inducible factor 1 (HIF-1), in a hypoxic condition and with nutrient deprivation, determines AXL transcription and activation of its signaling cascades (Figure 2A) [40,41].

Several microRNAs (miRNAs), downregulated or completely suppressed in several solid tumors, such as breast, lung and colorectal cancers, negatively affect AXL. Specifically, miR-34a and miR-199a/b bind AXL’s 3′UTR region and result inversely correlated with receptor levels (Figure 2B) [42]. A recent pre-clinical study showed that in breast cancer, AXL expression is essentially regulated by miR-34a, which promotes the repressive activity only in selective genetic contexts. The authors, indeed, have evaluated miR-34a levels in breast cancer cell lines with different genetic backgrounds: MDA-MB231 (TNBC cells) and SKBR3 (HER2-amplified cells). In both cases, miR-34a levels have resulted lower as compared to a normal breast cell context, but miRNA overexpression, mediated by genetic manipulation, decreased AXL levels only in TNBC cells [43].

#### 3.2.2. Post-Translational Modification

Proteolytic cleavage of extracellular domain represents, certainly, the most important post-translational modification of AXL. The process is mediated by the a disintegrin and metalloproteinase domain (ADAM) containing protein 10 and 17 and determines the formation of a soluble form of AXL (sAXL) that, binding GAS6, inhibits AXL activation (Figure 2C) [44,45]. The balance between sAXL and the receptor in plasma membrane would seem to be a regulation mechanism actuated in different cellular contexts. However, AXL activation occurs through different ways, which include its ability to interact with other membrane receptors [28]. This would explain why in many cancer contexts, despite the capture of GAS6 by sAXL, the activation of the receptor is still dysregulated. Because sAXL is particularly abundant in many tumors and is presumably associated with the expression levels of receptor in membrane, it is considered an important predictive biomarker in cancer progression and response to treatments [46,47]. A recent study has evaluated the presence of sAXL in the peripheral blood of patients with melanoma and showed a significant correlation between its high levels and reduced patient survival [47].

AXL’s stability and functionality are positively or negatively regulated by different proteins. Heat-shock protein 90 (HSP90), for example, is a molecular chaperonin which modulates the maturation and stabilization of many “client” proteins, such as AXL receptor (Figure 2C) [48,49]. In inflammatory breast cancer, AXL homeostasis is profoundly associated with Tazarotene-induced gene 1 (TIG1), a functionally undefined membrane protein, correlated with uncontrolled cell proliferation, migration and survival. TIG1 interaction with AXL simultaneously promotes the receptor stabilization in plasma membrane and activation of the nuclear factor kappa-light-chain-enhancer of activated B cells (NFκB) pathway and matrix metalloproteinase (MMP) 9, involved in cell progression and metastasis (Figure 2C) [50]. To the contrary, casitas B-lineage lymphoma (CBL) E3 ligases promote AXL degradation through a ubiquitination process [49,51,52]. In recent years, the attention of research has focused on the C1 domain-containing phosphatase and tensin homolog (C1-TEN) protein that, with a structure very similar to phosphatase and tensin homolog on chromosome 10 (PTEN), represents a new negative regulator of phosphatidylinositol 3-kinases (PI3K) signaling. Recent studies have shown that C1-TEN, by direct interaction with AXL’s catalytic subunit, inhibits the receptor ability to activate AKT, blocking cell survival signaling (Figure 2C) [53,54].

## 4. AXL Activation

### 4.1. GAS6/AXL Activation Mechanism

**Figure 3:**
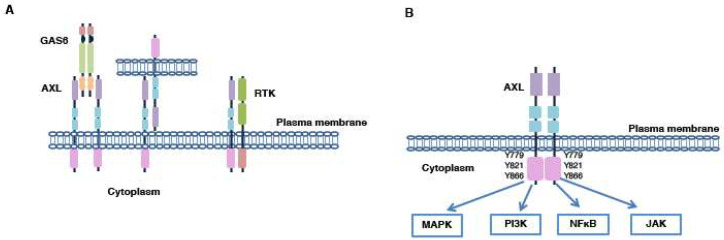
AXL activation. (**A**) AXL can be activated in several ways: (1) ligand-dependent dimerization; (2) ligand-independent activation mediated by interaction between two AXL monomers present on neighboring cells or with other members of the receptor tyrosine kinase (RTK) family. (**B**) AXL activation regulates many molecular pathways.

AXL/GAS6 interaction promotes a molecular complex formed by two molecules of AXL and GAS6 (2:2 stoichiometry) (Figure 3A). This activation mechanism results in trans-autophosphorylation of multiple tyrosine residues present in AXL’s cytoplasmic domain; three residues (Y779, Y821 and Y866) are involved in tumor development because their activation modulates several downstream effectors (Figure 3B). In particular, the Y779 and Y866 residues are implicated in PI3K and Phospholipase Cγ (PLCγ) activation, respectively; Y821 instead regulates multiple cytoplasmic proteins, such as PI3K, PLC, growth factor receptor-bound protein 2 (Grb2), c-Src and Lck [55].

In breast cancer, AXL’s activation, mediated by the link with GAS6, has been deeply studied, especially for its role in the connections between the tumor microenvironment (TME) and cancer cells. Indeed, it is known that macrophages’ production of GAS6 is the promoter of the receptor activation on tumor cells and GAS6 levels are often elevated in breast cancer models [56,57,58]. GAS6′s role as a negative prognostic factor, however, has been recently criticized by Ayman M. Ibrahim and collaborators. Tissue microarray studies have detected a higher GAS6 presence in ductal carcinomas in situ but not in metastatic forms. This clinical observation, therefore, highlighted the non-exclusive correlation between GAS6 and AXL in breast cancer progression [59].

### 4.2. GAS6-Independent Activation Mechanisms

As for the other RTKs, AXL is also activated by alternative mechanisms. Specifically, in breast cancer, AXL can be interact with its monomers present on neighboring cells or with other RTK family members (Figure 3A) [14,60,61,62,63,64]. For example, in TNBC tumors, AXL transactivates with epidermal growth factor receptor (EGFR), promoting drug resistance to inhibitors of the ErbB family [65,66]. TNBCs are characterized by high expression levels of both AXL and EGFR and this prerogative correlates with a poor prognosis. Only 20% of TNBC patients respond to EGFR inhibitors; indeed, the AXL/EGFR stable interaction activates AXL in a GAS6-independent manner and promotes the upregulation of many downstream effectors not activated by EGFR alone [67]. Co-immunoprecipitation experiments, conducted in TNBC contexts, have shown the formation of heterodimers composed of AXL and ErbB receptor family components, platelet-derived growth factor receptor (PDGFR) and hepatocyte growth factor receptor (MET) [67]. Particularly, AXL and MET cooperation, found in different tumor contexts, is correlated to increased tumor progression and migration. In response to hepatocyte growth factor (HGF), the two receptors cluster together in the plasma membrane, inducing the phosphorylation of the Y779 residue in the kinase domain of AXL and the activation of several molecular downstream targets regulated by AXL and MET [68]. These results support the idea that the simultaneous inhibition of several elements of the RTK family is necessary to bypass drug-resistance [67]. In HER2-amplified breast cancer, AXL heterodimerization with HER2 allows its GAS6-independent activation and promotes drug resistance and tumor progression. The use of murine models of HER2-positive breast tumors has shown, in a recent study, that the interaction between the two receptors stabilizes and activates AXL in membrane, favoring intravasation and extravasation phenomena. Moreover, the authors showed that the increase in AXL’s activity promotes a mesenchymal phenotype and is correlated with a poor prognosis for HER2-positive patients. AXL and HER2 pharmacological inhibition determines a reduction in metastasis in HER2-positive breast cancer mice [69].

## 5. AXL Implications in Breast Cancers

PI3K, mitogen-activated protein kinase (MAPK), Janus kinase (JAK) and NFκB pathways are the most important molecular cascades regulated by AXL. Their activation is implicated in many cellular processes related to carcinogenesis: migration, invasion, epithelial to mesenchymal transition (EMT), drug resistance, apoptosis and angiogenesis (Figure 3B) [23]. In particular, the PI3K pathway is dysregulated in various tumor forms, including breast cancer, and many drugs for its isoforms have been developed [70]. Several mutations of PI3K have been found in many malignancies, indicating their central role in the tumorigenic process [71].

The most important functions of AXL in breast tumors are summarized in Table 1.

### 5.1. AXL and Metastasis: Its Involvement in Cell Migration and Invasion

The cancer ability to invade distant organs and tissues promotes the formation of tumor colonies with new characteristics, making most treatments ineffective. In breast cancer, the metastatic process is finely regulated and AXL promotes cell invasion and migration [72,73,74]. Zhang and collaborators have shown, on 21 breast cancer cell lines with different invasive capabilities, that AXL mRNA is particularly expressed in cells with invasive and aggressive phenotypes. The authors, by altering the expression of the receptor by genetic or pharmacological manipulation, have observed changes in migratory capacity in all breast cancer cells analyzed. The tumor cells in which AXL was downregulated lost their invasive and migratory characteristics [75,76].

To migrate, cells need to polarize and rearrange the actin cytoskeleton. In mesenchymal TNBC tumors, an interesting study analyzed AXL’s direct involvement in cell polarization. AXL downregulation in two mesenchymal TNBC cell lines (MDA-MB231 and Hs578t) resulted in a cell motility decrease. Through immunofluorescence analysis, the authors showed a minimal presence of AXL around the plasma membrane in non-polarized cells; however, polarization increased the AXL levels, especially in cells’ anterior edge and at the Golgi apparatus. In this condition, AXL induced cytoskeleton reorganization by co-localizing with F-actin and by controlling actin polymerization. Indeed, it is known that one of the many downstream effectors of AXL is the small GTPase protein RAC, involved in actin polymerization and cell migration [77]. AXL pharmacological inhibition resulted in the blocking of its polarizing localization in TNBC mesenchymal cells. In order to further validate the results obtained in vitro, immunohistochemistry experiments were conducted on TNBC patients not responding to chemotherapy. These in vivo studies showed that although no heterogeneous expression of AXL was found in the tumor mass, it was still greater in tumor cells near the stroma [74]. The TME, especially represented by cancer-associated fibroblasts (CAFs), deeply influences cancer cells and their progression. CAFs which overexpress hMENAΔv6, an isoform of the actin-regulating protein hMENA, seem to secrete in the microenvironment with high levels of GAS6, the AXL ligand on tumor cells. At the same time, hMENAΔv6 promotes tumor receptor overexpression, supporting the GAS6/AXL axis involvement in tumor progression [78]. In a HER2-amplified breast context, AXL seems to play a key role in the metastatic process and its high levels are correlated with poor patient outcomes. Goyette et al. used mouse models to analyze AXL’s contribution to metastasis, suggesting a GAS6-independent receptor activation, mediated by HER2. Co-immunoprecipitation assays have validated this hypothesis both in genetically HER2-positive cells (SKBR3) and in contexts where HER2 is amplified by genetic manipulation (MCF10-A). Moreover, the authors have shown, in a model with AXL depletion, a significant decrease in its specific effectors, such as integrin, epidermal growth factor (EGF), Rho-GTPase and transforming growth factor β (TGF-β), as compared to cells with AXL. The inhibition of these pathways linked to AXL loss has blocked cellular migration and cytoskeleton reorganization [69]. Extracellular matrix (ECM) degradation is another mechanism that AXL supports to promote metastasis [50,79]. AXL overexpression increases MMP9 levels by activating the MAPK pathway; specific pharmacological inhibition of this pathway determines MMP9 expression levels reduction, but PI3K signaling inhibition does not produce significant effects. AXL transfection induces hyperactivation of NFκB and Brg-1, promoting the idea that these factors are all, together, responsible for AXL-mediated MMP9 activation [79]. Several studies have also shown that in order to perform this function, AXL’s activation is not mediated by interaction with GAS6 but benefits from its ability to transactivate with other membrane receptors [50,65,79].

According to the described functionalities, AXL can be considered an important mediator in the metastatic process; its high levels correlate with tumor stage and cancer progression and are essentially identified in distal metastasis [31,80]. A relevant study, conducted on a panel of 190 samples derived from patients with breast cancer, showed a significant correlation between AXL and reduced patient survival; in addition, clinical investigations have confirmed a higher expression of AXL in metastatic sites as compared to primary tumors of the same patients, confirming its involvement in tumor progression [81]. In vitro, AXL downregulation in MDA-MB231 cells significantly reduces their migratory and invasive capabilities and inhibits, in vivo, the formation of lung metastases [11]. Even in HER2-amplified contexts, AXL is the promoter of remote metastases. Goyette and collaborators have highlighted that high levels of AXL correlate with lung and brain metastases and poor prognosis [69]. The use of specific HER2-positive mouse models, presenting GAS6 or AXL losses, has confirmed the central role of this receptor in the metastatic process; indeed, the formation of lung metastases decreased in AXL^−/−^ mice [69]. The analysis, conducted in HER2-positive murine models described above, has also demonstrated that AXL is implicated in intravasation and extravasation phases of metastatic process. Indeed, AXL loss has promoted a significant reduction in circulating levels of tumor cells and has reduced their ability to exit blood vessels and colonize distant sites [69].

Steroid receptors (ER and PR), involved in tumor progression, are often unregulated in breast cancer [82]. Endocrine therapy has significantly improved the life expectancy of these patients by significantly increasing the OS [83]. The activation of steroid receptors generally occurs through the formation of stable protein complexes, such as with the SRC protein. Indeed, after stimulation, these receptors promote the active conformation of SRC, important for the proliferative response to hormones factors. The agents, able to antagonize the effect of hormones, block uncontrolled cell proliferation and promote cell cycle arrest [82]. In the context of hormone receptor-positive breast cancer, AXL’s role and its impact in the tumorigenesis has not yet been fully understood. Indeed, in recent years, several studies have shown controversial results. Berclaz and collaborators have suggested a significant correlation between AXL expression and ER-positive state. The analysis conducted on 111 patients showed that the expression of AXL in the membrane is significantly correlated (*p* < 0.0001) with the presence of ER, indicating a possible role of AXL as a mediator of the estrogenic action [80]. The opposite results were obtained from two independent studies in which either a non-correlation between AXL and ER or its correlation with the negative state of ER emerged [84,85]. A recent study, although it has not fully clarified the question, has partly confirmed the data of Berclaz et al. Indeed, through AXL expression analysis in 60 breast tumors and 40 benign breast lesions, its correlation with ER positivity has emerged [86]. Unlike what has been described in the literature so far, this study has also demonstrated a significant correlation between AXL levels and PR status [87].

In consideration of what has been described, the use of drugs able to mimic AXL loss could be a favorable pharmacological approach for patients already in disease progression. Pharmacogenomic analysis, conducted in TNBCs, has shown that a class of dopamine receptor antagonists mimics a condition similar to AXL depletion, resulting in tumor growth and invasive capacity reduction. Indeed, the AXL depletion gene expression signature in MDA-MB231 is comparable with the signature of these drugs. Dopamine receptor antagonists do not affect AXL functionality but block its downstream signaling: PI3K and MAPK pathways [88]. Although very interesting and innovative, this analysis will need to be further investigated. Indeed, the use of inhibitors at high concentrations on isolated cell lines can reasonably determine off-target phenomena, which are independent from the condition similar to AXL depletion.

### 5.2. AXL and EMT Process

EMT is the process by which cells, through the loss of their adhesion capacities, apical-basolateral polarity and epithelial markers, acquire the mesenchymal phenotype [89]. As described through in vitro experiments, EMT is promoted by several molecular factors, such as TGF-β, HGF and PDGF, or is mediated by Slug, Snail and Twist transcription factors [90,91]. Given its profound implications in cell migration and invasion, this process is commonly used by cancer cells to promote the metastatic process [91]. In breast cancer, cell transition to mesenchymal and invasive phenotypes is finely regulated, in several ways, by AXL [81]. Indeed, its high levels downregulate important pro-epithelial factors, such as E-cadherin and β-catenin, thus determining the acquisition of mesenchymal-like features of cancer cells [91]. Similarly, in vivo AXL upregulation and its activation are often associated to an increased expression of the most important mesenchymal markers (N-cadherin and Vimentin), resulting in poor prognosis for patients [92].

**Figure 4:**
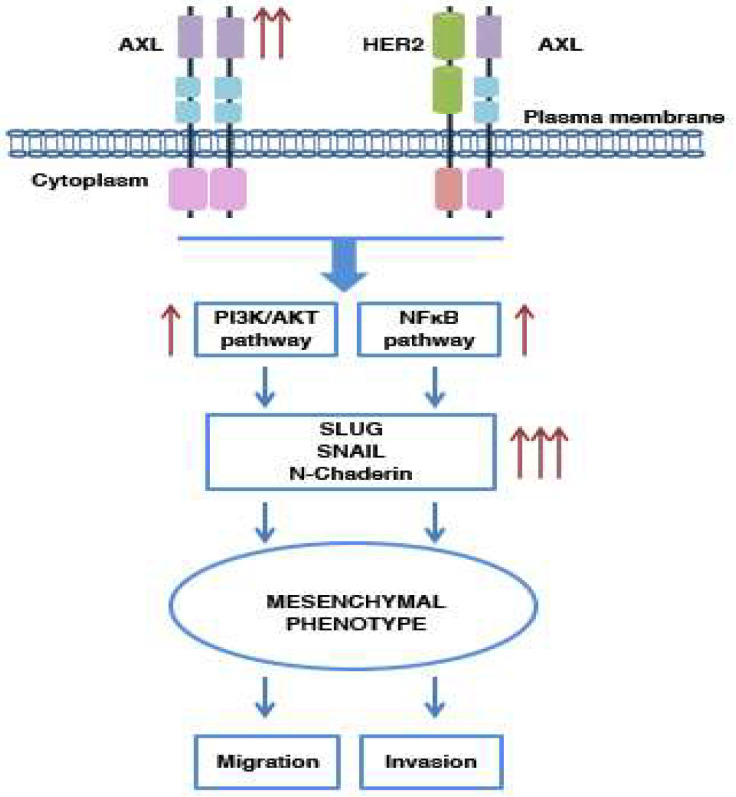
AXL involvement in the EMT process. In breast cancers, AXL is crucial for disease progression and is involved in the regulation of the EMT process. This receptor, when activated in a manner that is GAS6-dependent or not, induces the PI3K and NFkB pathways’ activation, implicated in cell mesenchymal state transition. These conditions determine an increase in migration and invasive properties of the breast cancer cells.

AXL-mediated activation of the PI3K and NFκB pathways promotes, in specific contexts, the increased expression of transcriptional EMT-positive regulator Slug and other EMT markers, such as N-cadherin [76,81,86,90]. Consistently, Slug is associated with aggressive forms of breast cancer, thereby highlighting AXL’s key role in tumor progression (Figure 4) [90].

Slug and Snail transfection into MCF10A (breast epithelial cells) promotes AXL overexpression and acquisition of a mesenchymal phenotype [81]. In HER2-positive breast tumors, TGF-β/AXL axis play an important role in tumor progression. Indeed, TGF-β promotes AXL expression in HER2-positive cells by increasing their invasive and migratory properties. AXL or TGF-β inhibition reverts this condition (Figure 4) [69].

AXL’s involvement in the EMT process is not limited to its function as a downstream effector, i.e., an effector activated only when the transition to a mesenchymal phenotype has to be promoted; in breast cancer stem cells (BCSCs), indeed, it also exerts a direct influence in this phenomenon. In BCSCs, AXL results constitutively active, independently of its link with GAS6, and it regulates the maintenance of cells in a mesenchymal state, determining an NFκB-mediated upregulation of N-cadherin, Snail and Slug [91]. These results are confirmed in MCF10A, genetically or pharmacologically manipulated for AXL expression. As expected, AXL inhibition leads to an increase in epithelial marker levels and reverts the EMT process [91].

### 5.3. AXL and Drug Resistance

AXL’s dysregulation promotes drug resistance in many cancer contexts and not only [36,67,93,94]. In TNBC tumors, AXL’s transactivation with EGFR or MET induces the formation of an intricate receptorial network able to increase the downstream signaling of molecular effectors which would not be activated, in the same way, by the isolated receptors [67]. Since AXL and MET are particularly similar in intracellular signaling, a simultaneous inhibition of the two receptors is desirable [95]. MAPK pathway inhibitors play the role of reprogrammers of RTKs activity. In TNBC cells, treatment with selective agents of this pathway determines a concomitant upregulation of AXL and HER2 levels as result of the simultaneous increase in their protein synthesis and degradation reduction. Therefore, a deep knowledge of the mechanisms involved in the reprogramming phenomena will better describe the hypothetical results of a treatment and will be able to better select drug combinations to overcome resistance [96]. In HER2- and ER-positive breast cancer cell lines, AXL promotes resistance to lapatinib and trastuzumab; for example, BT474 cells resistant to lapatinib show higher AXL levels as compared to the same control cells. A pharmacological downregulation of the receptor restores the response of these cells to lapatinib treatment, confirming AXL’s involvement in this process. Probably, the direct interaction between AXL and v-erb-b2 avian erythroblastic leukemia viral oncogene homolog 3 (HER3) overcomes the drug blockade of HER2 and determines the activation of its downstream pathways [97].

The suppression of immune response and the remodeling of the TME, especially by polarizing macrophages in the M2 subtype (pro-tumor subtype), are other mechanisms associated to AXL and tightly interconnected with drug resistance. In some in vivo experiments, conducted in mouse models, AXL pharmacological inhibition determines profound changes in TME organization and stimulates the antitumor immune response. Indeed, the receptor blockade maintains the macrophages in an M1 state and causes CD103^+^ dendritic cell activation in TME. This favorable condition induces cluster differentiation and (CD8)^+^ T lymphocytes’ proliferation and increases their antitumor activity [98,99,100]. Dysregulated activation of AXL can regulate the tumor release of paracrine factors involved in processes such as inflammation, migration and immune cell recruitment. AXL’s genetic or pharmacological depletion can, for example, influence the release of granulocytic-colony-stimulating factor (G-CSF), involved in the recruitment of granulocytic-myeloid-derived suppressor cells (G-MDSCs) in the TME [101,102]. In addition, the immunological microenvironment is deeply regulated and influenced by the GAS6/AXL axis. Several studies have shown that the inhibition of AXL promotes an anti-tumor microenvironment more responsive to therapies. Its depletion, indeed, leads to a significant reduction in many chemokines involved in the recruitment of monocytes and macrophages (such as C-C motif chemokine ligand (CCL)-2 and CCL-5). At the same time, this condition favors the production of molecules important for the attraction of CD8^+^ lymphocytes and natural killer (NK) cells (C-X-C Motif Chemokine Ligand (CXCL) 9, and CXCL10) [99,101]. A recent study by Christine Haider and collaborators has also demonstrated AXL’s role in the recruitment of neutrophils, often associated with poor prognosis. In hepatocellular carcinoma, the TGF-β/AXL axis promotes the release of CXCL-5, an attractive factor for these immune cells [103]. The mutual interconnections between the microenvironment and tumor cells influence the protein expression involved in tumor progression and drug resistance. During the early stages of mammary neoplasia development, macrophages induce tumor activation of survival pathways, promoting the transition to more invasive forms. It has been shown that GAS6, released by stromal cells, activates AXL and its molecular targets in cancer cells and causes alterations in E-cadherin expression [57]. An analysis conducted on pre-treatment melanoma biopsies has shown a higher receptor expression in patients with innate resistance to immunotherapy [104]. Moreover, the combination between TAM inhibitors and anti-PD-1, in a TNBC murine model, significantly decreased tumor growth and metastasis as compared to the monotherapy treatments and determined a major T cell infiltration [105]. Chimeric antigen receptor T (CAR-T) therapy shows important antitumor effects in patients with hematological malignancies but still remains ineffective in solid tumors. In vitro and in vivo experiments conducted in TNBC cells have shown encouraging results in terms of the antitumor activity of AXL-targeting CAR-T, confirming the real potential of this new therapeutic approach [106]. The promising results obtained in the preclinical field have led to the introduction of the pharmacological combination between AXL inhibitors and immunotherapy in various tumor contexts, including breast cancer, to bypass innate resistance. A recent phase II trial that involved the AXL inhibitor R428 in combination with pembrolizumab was concluded for TNBC patients, but the results of this study have not yet been disclosed [22]. A clinical trial (NCT03316586) characterized by nivolumab in combination with cabozantinib (another AXL inhibitor) for metastatic TNBC patients is now active [22].

## 6. AXL in BCSCs

Tumors are highly heterogeneous and well-organized structures, consisting of cell clusters with various differentiation statuses and biomarkers. BCSCs represent the most primitive cellular form, able to self-regenerate and maintain high plasticity by switching from an epithelial to a mesenchymal (and vice versa) state under TME influences [107]. BCSCs’ mesenchymal-like phenotype promotes invasion, metastasis and drug resistance and appears to be associated with AXL expression. In BCSCs, AXL, constitutively active (in a GAS6-independent manner), induces the EMT process through regulation of Slug, Snail and E/N-cadherin and is linked to the expression of important stem markers genes (Isl1, Cdc2a and Bglap1). AXL depletion promotes downregulation of the NFκB pathway and reverts the mesenchymal phenotype and pharmacological resistance of BCSCs [91].

## 7. AXL Inhibition in Breast Cancer Treatment

AXL inhibitors are classified, according to their action mechanisms, into three categories: (1) drugs that modulate and inhibit the kinase activity of the receptor; (2) specific antibodies that prevent the AXL dimerization and activation; (3) drugs that recognize and bind GAS6 [13].

The most important AXL inhibitors are summarized and described in Table 2.

### 7.1. AXL Selective Inhibitors

BGB324 (Bemcentinib or R428) is the more selective ATP-competitive inhibitor of AXL (IC_50_ = 14 nM) and was the first to enter clinical trials to treat several cancer forms, such as TNBC tumors, metastatic melanoma and NSCLC. By blocking AXL autophosphorylation on tyrosine residue Y821, in vitro, it induces apoptosis, inhibits cancer cell invasion and reverts erlotinib resistance in TNBC cells; in vivo, BGB324 reduces cancer metastasis [87,93,108,109]. In a recent preclinical study, BGB324 was tested in combination with auranofin, a gold phosphine derivative, initially used for the treatment of rheumatoid arthritis and also studied for the treatment of the other diseases, such as breast cancer. It is a thioredoxin reductase inhibitor and appears to induce apoptosis through PI3K pathway inhibition [110]. The authors of this study have observed that in different breast cancer settings (MDA-MB231 and MCF7), the combination of BGB234 and auranofin reduced cell growth by inducing apoptosis, mediated by increased levels of Bcl-2-associated X-protein (BAX) [111]. Recently, BGB324 was implicated in a phase II clinical trial (NCT03184558) for TNBC and inflammatory breast cancers, in combination with pembrolizumab; however, the results have not yet been released [22]. Other selective AXL inhibitors have been recently developed and have validated their efficacy in the preclinical field. NA80X-1 determines a decrease in cell motility and invasion in MDA-MB435 cells [75]. YW327.6S2 represents a potent anti-AXL monoclonal antibody that recognizes and binds AXL with high affinity. This antibody, by preventing the GAS6 interaction with the receptor, inhibits AXL activation and its downstream signaling [112]. GL21-T is an RNA-based aptamer which blocks AXL catalytic activity through interaction with the receptor extracellular domain [113]. In a recent preclinical study, the inhibitory action of this drug was evaluated in combination with the anti-metastatic miRNA148b. The authors have created a conjugate (AXL-148b) able to work only in positive AXL contexts. In vitro, by increasing the expression levels of miRNA148b, the conjugate has reduced the formation and mobility of mammospheres; in vivo, AXL-148b has blocked metastasis formation [114]. DN10764 (or AZD7762), developed as selective inhibitor of checkpoint kinases (ChKs) 1 and 2, is also involved in AXL downregulation. In a preclinical field study, DN10764 inhibited, in both vitro and in vivo experiments, AXL-dependent cell proliferation, invasion and migration and also induces apoptosis through caspases 3/7 activation [115,116]. SGI-7079 is another selective AXL inhibitor which, however, also targets the other members of the TAM family; in vitro experiments showed that it decreased cell proliferation and metastasis [50].

### 7.2. Multi-Targets Inhibitors

Rebastinib (or DCC-2036) is a multi-target inhibitor involved in the regulation of cell proliferation, invasion, migration and EMT processes by blocking the activity of several TKs, such as MET, vascular endothelial growth factor receptor 2 (VEGFR2), SRC and AXL. A recent in vitro and in vivo study has shown that in TNBCs (little responsive to hormonal and anti-HER2 therapies), rebastinib inhibited cell proliferation, invasion and EMT more efficiently as compared to other drugs used for breast cancer treatment. In addition, its combination with lapatinib or cisplatin significantly decreased the growth of TNBC cells [27]. Although it does not have a single site of action, rebastinib seems to carry out its inhibitory function mainly on AXL and its downstream targets. Indeed, the drug decreases cell growth only in murine models inoculated with TNBC characterized by high levels of AXL [117].

Cabozantinib (or XL184) recognizes and blocks many RTKs, such as Rearranged during Transfection (RET), VEGFR2, Kit, fms-related tyrosine kinases (Flt) 1, 3 and 4, tyrosine kinase with immunoglobulin-like and EGF-like domains 2 (Tie2), MET and AXL [29]. It is implicated in the treatment of several solid malignancies, such as renal cell carcinoma, medullary thyroid, NSCLC and TNBC tumors, by decreasing the metastatic and invasive potential of cancer cells. In a recent phase II trial (NCT01738438) conducted on metastatic TNBC patients, cabozantinib administration led to a clinical benefit of 34%, determining a median PFS of 2.0 months; moreover, cabozantinib treatment has determined quite encouraging results in terms of immune system activation. Indeed, patients treated with this drug showed higher circulating levels of CD8^+^ T lymphocytes and a greater activation of antitumor immunity. However, these results are still not very consistent and need further investigation [118,119,120,121,122].

Foretinib (XL880 or GSK-1363089) is another multi-kinase inhibitor which blocks AXL, MET, RET and VEGFR2 activity. In HER2-amplified breast tumors, it restores sensitivity to lapatinib and trastuzumab in resistant cells with high levels of AXL. AXL and HER3 interaction bypasses the lapatinib-mediated HER2 block, promoting PI3K/AKT pathway hyperactivation and drug resistance. AXL inhibition, mediated by foretinib, removes the AXL/HER3 interconnection and restores cell response to lapatinib [97]. In a phase II clinical trial (NCT01147484), 46% of enrolled TNBC patients benefited from foretinib treatment [22,130].

Merestinib (or LY2801653) is a small molecule kinase inhibitor which targets AXL, MET, macrophage-stimulating protein receptor (MST1R) and MAP kinase-interacting serine/threonine kinase 1 (MKNK1/2). In vitro and in vivo experiments have shown that merestinib inhibits angiogenesis and mitosis [123,124].

Bosutinib (or SKI-606), originally identified as an SRC and Abelson murine leukemia viral oncogene (Abl) kinase inhibitor, is also a powerful inhibitor of AXL, Mitogen-activated protein kinase kinase (MEK) and BMX [125]. In breast cancer, it regulates invasion, metastasis and tumor differentiation [126,127]. Bosutinib is implicated, for breast tumors, in several clinical trials [22].

Crizotinib (or PF-02341066) is an ATP-competitive small-molecule inhibitor which blocks MET, anaplastic lymphoma receptor tyrosine kinase (ALK), c-ros oncogene 1 receptor tyrosine kinase (ROS1) and AXL. In breast cancer cell lines, it inhibits cellular proliferation [128,129]. As described for the other non-selective inhibitors, crizotinib is also involved in several clinical trials as a modulator of other molecular targets [22].

## 8. AXL Clinical Implications: Limits and Future Hopes

The clinical management of cancer patients has been profoundly transformed in recent decades. The use of targeted therapies and immunotherapy has revolutionized the clinical course of many cancers, including breast cancer. These new therapeutic scenarios, however, especially in tumor contexts which lack effective long-term treatment (e.g., TNBC tumors), require the continuous study of new biomarkers. AXL seems to be an optimal candidate for the development of new therapeutic approaches. Indeed, it is implicated in the various stages of tumorigenesis and it is considered an important modulator of the TME. Currently consolidated in hematological malignancies and rapidly evolving even in solid tumors, anti-AXL therapies are continuously enriched with new inhibitors [12,131]. However, the high structural affinity of AXL with other RTKs does not make life easy for researchers; indeed, most of the AXL inhibitors involved in clinical trials are multi-target and are considered primarily for their action on other sites. In addition, many multi-target agents (also in use for the treatment of breast cancer), because of their low selectivity, have a difficult clinical management and are often associated with toxicity events [118,130]. In addition, the idea of a pharmacological blockade of GAS6 has important limits because, as widely described, AXL can activate itself independently from its ligand. In addition, in breast cancer, it has been recently demonstrated that GAS6 levels, elevated in carcinoma in situ, decrease dramatically in the most invasive forms of cancer. This result further confirms that the functions of GAS6 and AXL can work independently from each other [59]. To date, BGB324 is the only AXL-specific inhibitor to be involved in clinical trials, although other highly selective molecules have been developed. Of these, however, there are only promising preclinical data which need to be extensively investigated [75,111,112]. It is, therefore, clear that the process of development and the testing of new selective AXL inhibitors will require more time and more extensive studies, in the clinical field also. Analyzing the results described in this review, it is clear that AXL can be a potential marker in which to invest for breast cancer management and not only this, but there is still no solid and definitive evidence on its role in the clinical field. For this reason, it is auspicious to increase the study of selective inhibitors for AXL that can also be combined with other therapeutic regimens.

## 9. Conclusions

In recent years, cancer treatments have made important progress and the discovery of new target therapies has greatly improved the lives of patients and survival rates. However, despite the promising results obtained, oncology research has not solved two problems that are the causes of the most cancer deaths: (1) metastasis, with new characteristics as compared to the primary tumor; (2) drug resistance. For these reasons, it is essential to identify and study, always, new and alternative molecular targets to bypass these unfortunate conditions. Given its highly oncogenic characteristics and its involvement in many pro-tumorigenic processes, currently, AXL is considered a valid biomarker in several tumor contexts, primarily breast cancer. In this pathology, through uncontrolled activation of different downstream effectors, such as PI3K and MAPK, AXL regulates the malignant progression and migratory properties of breast cancer cells. Especially in TNBC tumors, AXL has been considered a key factor in the EMT process, promoting a mesenchymal and invasive phenotype. No less important is the role of AXL in several mechanisms of pharmacological resistance involved in the disease’s relapse, even in contexts previously responding to treatments. To date, several agents which inhibit the GAS6/AXL axis are being studied and have led to important results from both in vitro and in vivo experiments. Certainly, BGB324, due to its high selectivity for AXL, is the drug that has mainly produced significant results in terms of growth inhibition; in addition, it is the only specific AXL inhibitor to be involved in clinical trials for the treatment of several cancers. The development of new anti-AXL agents is always active, but even if preclinical studies have produced encouraging results in multiple tumor contexts, there is still a long way to go in clinical trials. Indeed, although different inhibitors of AXL have been developed, many of these are multi-target and are often implicated in clinical trials not for their inhibitory role on AXL. Therefore, the construction of new drugs, increasingly selective for this receptor, is always expected.

## Figures and Tables

**Table 1:** AXL involvement into tumorigenesis. Tumor microenvironment (TME); extracellular matrix (ECM); epithelial–mesenchymal transition (EMT); receptor tyrosine kinase (RTK).

AXL Function(s)	
**Tumor progression and metastasis**	Rearrangement of the actin cytoskeleton Involvement in cell polarization Mediator between tumor cells and TME ECM degradation Involvement in intravasation and extravasation phenomena
**EMT process**	↓E-cadherin and β-catenin ↑N-cadherin, Vimentin and Slug
**Drug resistance**	Transactivation with other members of RTK family Polarization of macrophages into M2-subtype Induction of a pro-tumoral immune microenvironment

**Table 2:** AXL inhibitors. Several AXL inhibitors (selective or not) are involved in the treatment of breast cancer. Many of them, however, are still in preclinical development.

Selective Inhibitors				
	Target(s)	Function(s)	Field(s)	Reference(s)
BGB324 (Bemcentinib or R428)	AXL	ATP-competitive AXL inhibitor that promotes apoptosis and reduces cell growth and metastasis	Preclinical and clinical	[87,93,108,109,110,111]
NA80X-1	AXL	Selective AXL inhibitor that decreases cell motility and invasion	Preclinical	[75]
YW327.6S2	AXL	Monoclonal antibody that inhibits GAS6/AXL interaction	Preclinical	[112]
GL21-T	AXL	RNA-based aptamer that blocks AXL’s catalytic activity and inhibits mobility and metastasis	Preclinical	[113,114]
DN10764 (AZD7762)	AXL ChKs	Selective AXL inhibitor that decreases cell proliferation, invasion and migration and induces apoptosis	Preclinical	[115,116]
SGI-7079	AXL	Selective AXL inhibitor that decreases cell proliferation and metastasis	Preclinical	[50]
**Non-Selective Inhibitors**				
Rebastinib (DCC-2036)	MET VEGFR2 SRC AXL	Multi-target inhibitor that decreases cell proliferation, invasion, migration and EMT	Preclinical and Clinical	[27,117]
Cabozantinib (XL184)	RET VEGFR2 Flt 1-3-4 Tie2 MET AXL	Multi-target inhibitor that decreases cellular invasion and promotes immune system activation	Preclinical and clinical	[29,118,119,120,121,122]
Foretinib (XL880 or GSK-1363089)	MET RET VEGFR2 AXL	Multi-target inhibitor that restores the response to lapatinib in HER2+ context	Preclinical and clinical	[97]
Merestinib (LY2801653)	MET MST1R MKNK1/2 AXL	Multi-target agent that inhibits angiogenesis and mitosis	Preclinical and clinical	[123,124]
Bosutinib (SKI-606)	SRC Abl MEK BMX AXL	Multi-target inhibitors that decreases invasion, metastasis and tumor differentiation	Preclinical and clinical	[125,126,127]
Crizotinib (PF-02341066)	MET ALK ROS1 AXL	ATP-competitive agent that inhibits cell proliferation	Preclinical and clinical	[128,129]

## Abbreviations

The following abbreviations are used in this manuscript:

Abl	Abelson murine leukemia viral oncogene
ADAM	Disintegrin and metalloproteinase domain-containing protein
ALK	Anaplastic lymphoma receptor tyrosine kinase
AP-1	Activator protein 1
BAX	Bcl-2 Associated X-protein
BCSC	Breast cancer stem cell
C1-TEN	C1 domain-containing phosphatase and tensin homolog
CAFs	Cancer-associated fibroblasts
CAR-T	Chimeric antigen receptor T
CBL	Casitas B-lineage lymphoma
CCL	C-C motif chemokine ligand
CD	Cluster differentiation
ChK	Checkpoint kinases
CXCL	C-X-C Motif Chemokine Ligand
ECM	Extracellular Matrix
EGF	Epidermal growth factor
EGFR	Epidermal growth factor receptor
EMT	Epithelial–mesenchymal transition
ER	Estrogen Receptor
FLT	fms-related tyrosine kinase
FNIII	Fibronectin type III
FRA-1	Transcription factor Fos-related antigen 1
GAS6	Growth arrest-specific protein 6
G-CSF	Granulocytic-colony-stimulating factor
G-MDSC	Granulocytic-myeloid-derived suppressor cell
GLA	Gamma-carboxy-glutamic acid
GRB2	Growth factor receptor-bound protein 2
HER2	Human epidermal growth factor receptor 2
HER3	V-erb-b2 avian erythroblastic leukemia viral oncogene homolog3
HGF	Hepatocyte growth factor
HIF-1	hypoxia inducible factor 1
HSP90	Heat-shock protein 90
Ig	Immunoglobulin
JAK	Janus Kinase
LG	Laminin G-like
MAPK	Mitogen-activated protein kinase
MEK	Mitogen-activated protein kinase kinase
MET	Hepatocyte growth factor receptor
MicroRNA	miRNA
MKNK1/2	MAP kinase-interacting serine/threonine kinase 1
MMP9	Matrix metalloproteinase 9
MST1R	Macrophage-stimulating protein receptor
MZF-1	Myeloid Zinc Finger 1
NFκB	Nuclear factor kappa-light-chain-enhancer of activated B cells
NSCLC	Non-small-cell lung cancer
OS	Overall survival
PDGFR	Platelet-derived growth factor receptor
PI3K	PhosphoInositide3-Kinase
PLC γ	Phospholipase C γ
PR	Progesterone receptor
PROS1	Protein S
PTEN	Phosphatase and tensin homolog on chromosome 10
RET	Rearranged during Transfection
ROS1	c-ros oncogene 1, receptor tyrosine kinase
RTKs	Receptor tyrosine kinases
sAXL	Soluble AXL
SNPs	Single polymorphisms
SP	Specificity protein
TAM	TYRO-3, AXL and MER
TGF-β	Transforming growth factor β
TIE-2	Tyrosine kinase with immunoglobulin-like and EGF-like domains 2
TIG1	Tazarotene-induced gene 1
TKs	Tyrosine kinases
TNBC	Triple-negative breast cancer
TULP-1	Tubby-like protein 1
VEGFR2	Vascula Endothelial Growth Factor Receptor 2
YAP1	Yes-associated Protein 1
